# Hepatoprotective Activity of Nature-Derived Polyphenols Studied by Mass Spectrometry Based Multi-OMICS Approach

**DOI:** 10.3390/ijms26041604

**Published:** 2025-02-13

**Authors:** Alexander G. Brzhozovskiy, Savva D. Semenov, Alexander Y. Zherebker, Anna E. Bugrova, Maria N. Yurova, Yury V. Zhernov, Oxana A. Kovaleva, Alexander L. Semenov, Dmitry P. Abroskin, Stepan S. Kruglov, Elena I. Fedoros, Alexey S. Kononikhin, Evgeniy N. Nikolaev

**Affiliations:** 1 Project Center of Omics Technologies and Advanced Mass Spectrometry, 121205 Moscow, Russia; roporoz@gmail.com (S.D.S.); zherebker@gmail.com (A.Y.Z.); yumarni@gmail.com (M.N.Y.); zhernov@list.ru (Y.V.Z.); okovaleva504@gmail.com (O.A.K.); mr.mantikor@gmail.com (D.P.A.);; 2 Moscow Center for Advanced Studies, 123592 Moscow, Russia; 3 Emanuel Institute of Biochemical Physics, Russian Academy of Sciences, 119334 Moscow, Russia; 4 N.N. Petrov National Medical Research Center of Oncology, 197758 Saint Petersburg, Russia; genesem7@gmail.com (A.L.S.); oncokss@gmail.com (S.S.K.);; 5 A.N. Sysin Research Institute of Human Ecology and Environmental Hygiene, Centre for Strategic Planning and Management of Biomedical Health Risks of the Federal Medical and Biological Agency, 119435 Moscow, Russia; 6 Department of General Hygiene, F. Erismann Institute of Public Health, I.M. Sechenov First Moscow State Medical University (Sechenov University), 119435 Moscow, Russia

**Keywords:** proteomics, liver, mice, biomarkers, mass spectrometry

## Abstract

The aim of this study was to examine the hepatoprotective activity of multicomponent mixtures of natural origin in the BALB/C mouse model, with subacute liver failure (SALF) induced by the administration of toxin carbon tetrachloride (CCl_4_). The hepatoprotective activity of activated hydrolytic lignin (BP-Cx-1), humic acid peloids (HA), and isoflavones from kudzu Pueraria lobata roots (IFL) was evaluated using mass spectrometry (MS)-based omics technologies. Our MS-based approach revealed new insights into the molecular mechanisms of the hepatoprotective activity of multicomponent mixtures of natural origin. Significant differences were observed in the proteome and metabolome profiles of the urine and liver of BALB/c mice with SALF between a control group with CCl_4_ administration, intact controls, and groups receiving potential hepatoprotectors of natural origin (BP-Cx-1, HA, IFL). Proteomic and metabolomics analyses demonstrated that among the hepatoprotectors, IFL possessed the highest hepatoprotective potential, which correlated well with the relative effectiveness of the drugs recorded during in vitro studies. These results correlate with the relative effectiveness of the drugs recorded in previous in vitro and in vivo studies. The leading IFL activity may be attributed to a higher content of active polyphenolic components compared to heterogeneous HA and BP-Cx-1. Enrichment with active components by fractionation is a direction that can be explored for developing hepatoprotective agents based on natural complex polyphenols.

## 1. Introduction

Natural-origin complex mixtures and their derivatives possess a wide range of biological activities and, often, low toxicity, which makes them promising candidates for therapeutic applications. The pharmacological effects of nature-derived mixtures have been demonstrated in numerous studies [1,2]. Antioxidant activity, which is inherent to polyphenolic compounds, is believed to be the leading mode of action of lignin-like compounds [3]. Polyphenols are promising biologically active compounds with antioxidant and anti-inflammatory properties [4,5]. Natural agents containing phenolic sub-structures have demonstrated the ability to inactivate free radicals such as superoxide anions [6]. Lignin-based products have demonstrated antimutagenic and anticarcinogenic activity associated with their sorption capacity and antioxidant properties; in particular, a significant reduction in DNA strand breaks was shown in a DNA comet assay of hamster lung V79 cells and human colon Caco-2 cells exposed to N-methyl-N′-nitro-N-nitrosoguanidine [7]. Notably, the pharmacological effects of lignin derivatives are quite wide. They are considered agents for the treatment of diabetes (lignosulfonic acid) [8], obesity (lignophenols) [9], HIV infection (lignosulfonic acid, lignin-carbohydrate complexes) [10], and emphysema and as anticoagulants (sulfated low molecular weight lignins) [11] and potential nano-drug delivery systems [12]. However, lignin-based products are characterized by a low bioavailability in animals due to its poor solubility in water [13]. Nowadays, hydrolytic lignin is suggested as an adsorbent for acute poisoning with drugs, alcohol, salts of heavy metals, alkaloids, dysentery, dysbacteriosis, dyspepsia, foodborne toxic infections, salmonellosis, intoxications associated with purulent inflammatory diseases, liver and renal failure, food and drug allergies, etc. [14,15]. Liquid-phase hydrolysis has been used for preparing water-soluble lignin derivatives with higher bioavailability, resulting in the water-soluble modification of lignin—BP-Cx-1 [3]. One of the fundamental differences between BP-Cx-1 and other polyphenolic mixtures is its low degree of oxidation, high content of aromatic structures, and the absence of nitrogen- and sulfur-containing components [3]. Humic substances (HSs) and isoflavones are another class of complex mixtures with bioactive molecules of natural origin with potent antioxidant properties. HSs comprise a large variety of compounds including lipids, tannins, carbohydrates, and lignins, and show a wide spectrum of biological activity such as antiviral activity [15,16]. Previously, it was shown that HSs can inhibit class A β-Lactamase (TEM-1) [17]. Isoflavones are structurally similar to estradiol-17β and have molecular structures similar to animal estrogens, which can suppress excessive immune cell activation and delay hypersensitivity [18]. The pharmacological activity of the abovementioned products can be attributed mostly to the presence of polyphenolic moieties. Nevertheless, their unique synergetic effects make natural mixtures promising, reproducible candidates for drug development. The area of biomedical applications of natural origin complex mixtures and their derivatives remains actively growing. Mass spectrometry (MS)-based omics methods—in particular, high-resolution MS techniques—enable the characterization of the wide, multicomponent compositions of complex mixtures, and identify potential bioactive natural drug compounds and the molecular mechanisms of their action [19]. In our previous study, active components were elucidated by the extraction of D-labeled BP-Cx-1 from mice livers followed by MS analysis, and potential compounds with hepatoprotective properties were identified [20]. The penetration of particular components in liver tissue indicated the existence of bioactive pool of molecules in complex mixtures and the potential to use them to treat disorders that lack existing drugs, including liver damage.

Subacute liver failure (SALF) is a complication of acute hepatitis (AH), a rare disorder defined by the clinical progression of jaundice to hepatic encephalopathy in the absence of pre-existing liver disease in fewer than 28 days [21]. SALF can be associated with seronegative hepatitis, drug-induced liver failure, autoimmune hepatitis, Budd–Chiari syndrome, and Wilson’s disease [22]. AH usually causes a high mortality risk or increased tendency to cirrhosis development [23]. SALF’s pathogenesis is unknown and its treatment is unsatisfactory [23]. Once the criteria for poor AH prognosis have been met, the only effective treatment is liver transplantation [21].

The aim of this study was to examine the hepatoprotective activity of multicomponent mixtures of natural origin in the BALB/C mice model with SALF induced by the toxin carbon tetrachloride (CCl_4_). The hepatoprotective activity of activated hydrolytic lignin (BP-Cx-1), humic acid peloids (HA), and isoflavones from kudzu Pueraria lobata (IFL) roots was evaluated using mass spectrometry-based omics technologies.

## 2. Results

### 2.1. Overview of the Study Pipeline

**Figure 1:**
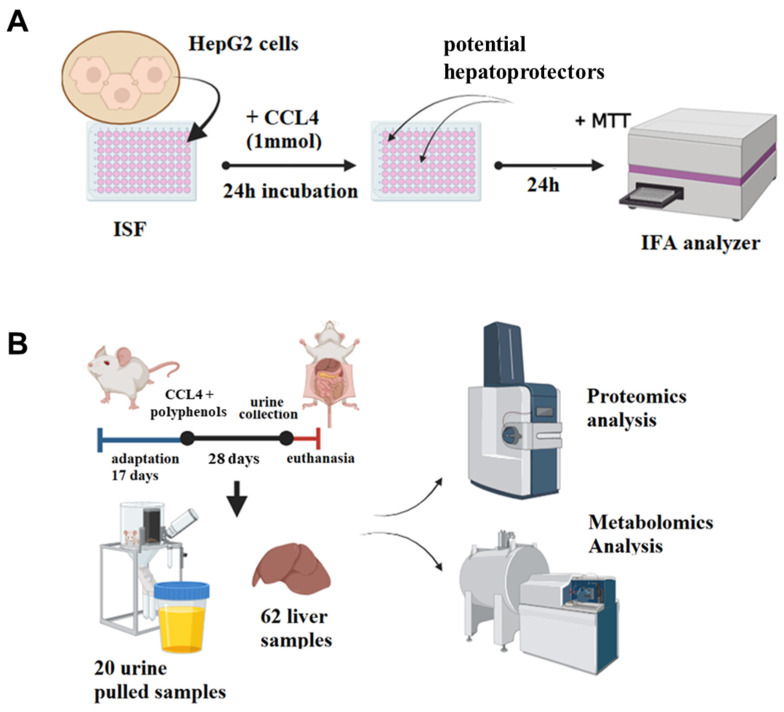
The study pipeline used in this work. (**A**) An MTT colorimetric assay was used to determine the cell cytotoxicity and hepatoprotective activity of multicomponent mixtures of polyphenolic compounds on a HepG2 hepatocyte cell culture. (**B**) In 62 female BALB/C mice, subacute liver failure was modeled by CCl_4_ i.p. injections (1 mL/kg, ×6), and various polyphenolic substances were intragastrically administered for 28 days. Liver and urine samples were collected and analyzed using an LC–MS method to reveal alterations in their protein and metabolite composition.

The study included five samples extracted from natural sources and one synthetic mixture reported in our previous work [20]: fractions of humic acids (HA), fulvic acid (FA), castoreum extract (CE) isoflavones from kudzu Pueraria lobata roots (IFL), and the water-soluble lignin derivative BP-Cx-1. To examine the hepatoprotective activity of natural and nature-derived polyphenols, we developed the following methodology (Figure 1): In the first step, the cell cytotoxicity and hepatoprotective activity of the investigated mixtures were determined on a HepG2 hepatocyte cell culture. Secondly, samples with a pronounced hepatoprotective effect were included for in vivo testing in a subacute liver failure model in BALB/C mice. The in vivo study included the collection of urine and liver tissues for proteome analysis with LC–MS, and non-target analysis of metabolites with ultra-high resolution mass spectrometry.

### 2.2. Hepatoprotective Effect In Vitro

**Figure 2:**
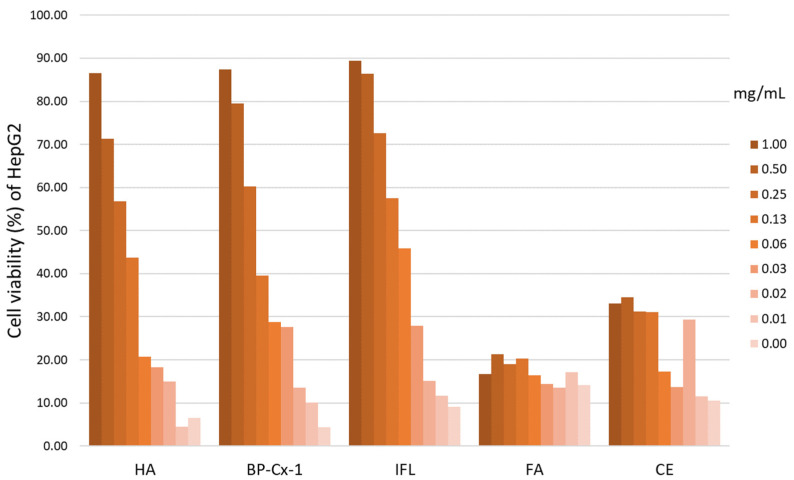
MTT colorimetric assay results. Cell viability (%) of HepG2 cells was plotted against the concentration (mg/mL) of potential hepatoprotective agents.

An MTT colorimetric assay was conducted to determine the hepatoprotective effects of nature-derived polyphenols (%), on average across two repetitions. HepG2 hepatocyte cell cultures were incubated in a therapeutic mode, with the addition of a hepatoprotector to the culture medium after incubation of hepatocytes with CCl_4_. Samples included humic (HA) and fulvic acid (FA), castoreum extract (CE), BP-Cx-1, and isoflavones from kudzu Pueraria lobata roots (IFL). HA, IFL, and BP-Cx-1 showed a pronounced hepatoprotective effect (Figure 2, Table S1) and were selected for further in vivo study. Cell viability was identified by measuring the optical density with a microplate reader at 630 nm. For all samples, the effective dose 50 (ED50) was calculated using linear–logarithmic interpolation using the AAT Bioquest online calculator: ED50 HA = 238.9997 μg/mL; ED50 BP-Cx-1 = 236.9988 µg/mL; ED50 IFL = 87.1657 µg/mL (Figure S1).

BP-Cx-1, HA, and IFL were included for in vitro cell cytotoxicity tests on Salmonella typhimurium (strain TA100). In comparison with the negative control, the significance of the paired comparisons was 0.8 < *p* < 0.9, which is more than *p* = 0.05. This substantiates the proposed null hypothesis concerning the absence of statistically significant differences between the negative control and experimental variants in the number of revertant colonies.

Based on the obtained results, we concluded that the tested concentrations of the mixtures of natural origin (HA, BP-Cx-1, and IFL) do not have a pronounced toxic effect on the tested *S. typhimurium* TA 100 strain. The survival rate of bacteria in the experimental variants varied within the range of 98.1–100.0%.

### 2.3. Hepatoprotective Effects In Vivo

The dynamics of the body weight of the mice in the model of subacute liver failure induced by CCl_4_ are presented in Table S2. Statistically significant differences in the body weight of animals in the experimental groups compared to the negative control group were revealed in the CCl_4_ group, as well as in the BP-Cx-1 group. On the 17th day after the first administration of CCl_4_, a significant decrease in body weight was observed in the CCl_4_ group, reaching 20.01 ± 1.56 g (vs. 21.24 ± 1.47 g in the negative control group (*p*-value < 0.05). At 3, 7, 10, 14, 17, 21 days after the first administration of CCl_4_, the body weight in group CCl_4_ + BP-Cx-1 also decreased statistically significantly relative to the values of the negative control (*p*-value < 0.05). The data on the body weight dynamics of the experimental animals indicate the presence of toxic effects in all groups where CCl_4_ was administered, which corresponds to the literature data and data from previous experiments conducted at our base using CCl_4_ [24]. Also, significant changes compared to the CCl_4_ group were revealed in the CCl_4_ + BP-Cx-1 group on the 14th day. In connection with the data obtained on the dynamics of body weight of experimental animals, we can conclude that the weight gain was less pronounced in the CCl_4_ + BP-Cx-1 group.

The liver mass index in the negative control group (5.4 ± 0.11) was significantly lower than in all CCl_4_-induced pathology groups, except for the group with IFL (5.6 ± 0.07, *p* = 0.7403) (Figure S2). Treatment with IFL significantly decreased the mass index of the liver in comparison with the CCl_4_ group (6.0 ± 0.09, *p* = 0.02). HA and BP-Cx-1 treatment slightly decreased the liver mass index without statistical significance (5.96 ± 0.11, *p* = 0.9997 and 5.83 ± 0.13, *p* = 0.7118, respectively).

**Figure 3:**
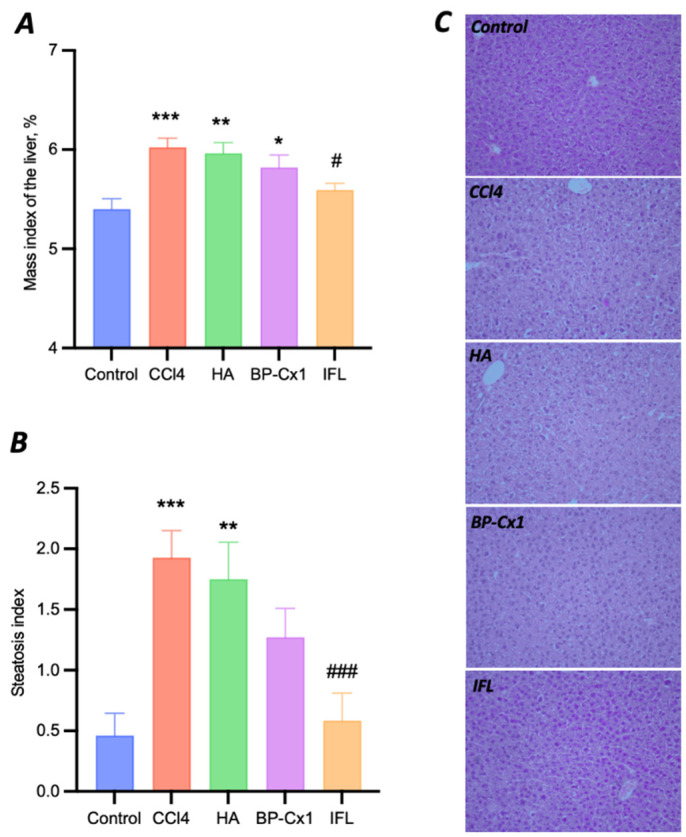
Data on the evaluation of the hepatoprotective activity of naturally derived compounds in a model of CCl_4_ liver toxicity in BALB/C mice. Mass index of the liver (percentage ratio of liver mass to body weight) (**A**), steatosis index (**B**), and micro photos of histological slides of the liver ×200 (**C**). Data (**A**,**B**) are M ± SE. Differences are statistically significant to the Control group: ***—*p* < 0.001, **—*p* < 0.01, *—*p* < 0.05; to the CCl_4_ group: ###—*p* < 0.001, #—*p* < 0.05.

All mice treated with CCl_4_ developed severe microvesicular hepatic steatosis. In this liver injury model, the steatosis index was increased four-fold by CCl_4_ as compared to the negative control (1.93 ± 0.22 and 0.46 ± 0.18, respectively, *p* = 0.0002). IFL treatment showed pronounced hepatoprotector activity, significantly decreasing the steatosis index by 70% to 0.58 ± 0.23 (*p* = 0.0008 vs. CCl_4_ and *p* = 0.8300 vs. Control) (Figure 3). The steatosis index was lower in the CCl_4_ group than in the BP-Cx-1 group by 34% (1.27 ± 0.24, *p* = 0.1574 vs. CCl_4_ and *p* = 0.0805 vs. Control) and in the HA group by 11% (1.75 ± 0.3, *p* = 0.8300 vs. CCl_4_ and *p* = 0.0014 vs. Control). Thus, macro and micro studies of the liver showed subacute liver failure development by CCl_4_ and the hepatoprotective activity of selected agents. Moreover, the results of in vitro and in vivo tests corroborated well, with IFL demonstrating the highest activity.

### 2.4. Results of Proteomic Analysis

#### 2.4.1. Urine Proteome Fraction Analysis

In the results of the proteomic analysis of the 20 urine pooled samples collected with metabolic cages, 1882 protein groups were identified (FDR 1%) (Table S3). Evaluation of the proteomic composition in the different groups was performed via hierarchical clustering based on the average levels of label-free quantification (LFQ) intensities. Semi-quantitative label-free proteomic analysis revealed 47 proteins (*p*-value < 0.01) for which the relative abundances were significantly changed in the CCl_4_ group with respect to the negative control (Table S4). Enrichment analysis was conducted via the GO database using STRING software v.12 [25]. The proteins significantly changed in the CCL_4_ group are involved in extracellular matrix organization (Comp, Ctsb, Vcam1, Spp1, Ceacam1, Ctsl), as well as in abnormal wound healing (Scpep1, Comp, Tgfbr1, Spp1, Ceacam1).

Multisample ANOVA analysis of the BP-Cx-1 and CCL_4_ groups with respect to the negative control group resulted in 117 significantly changed protein groups. These proteins participate in extracellular matrix organization (Cdh1, Icam2, Comp, Nid1, Ctsb, Plg, Vtn, Madcam1, Vcam1, Pcolce, F11r, Fn1, Efemp2, Spp1, Ceacam1, Cd47, Ctsd), abnormal vascular wound healing (Comp, Lrp1, Spp1, Ceacam1), and in innate immune system pathways (Gm2a, Icam2, Ctsb, Vtn, Npc2, Ly86, Plau, Clu, Cfb, Dpp7, Dsc1, Gns, Hp, Cfi, Ggh, Ceacam1, Ear1, Cd47, Creg1, Ctsd, Ear6, Rnaset2b, Psap, Mme).

#### 2.4.2. Liver Proteome Fraction Analysis

**Figure 4:**
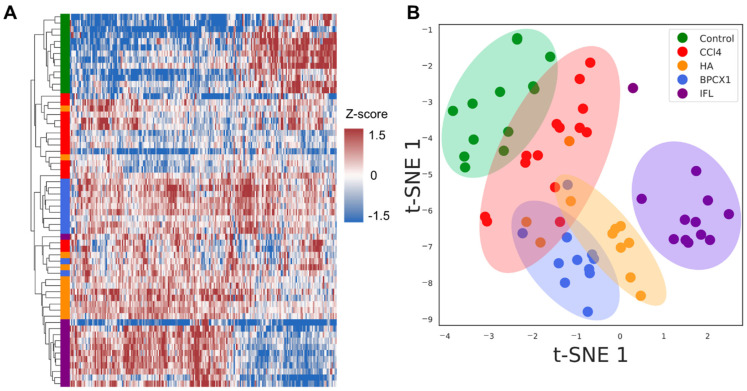
(**A**) Heat map of the significantly changed liver proteins (FDR < 0.01) based on the z-scores of the normalized LFQ values. The strength of the colors indicates the relative abundance of the protein in different groups. (**B**) Clusterization by t-SNE of liver proteomic data for all 62 samples. Colored dots depict the liver samples: Control—green; CCl_4_—red; HA–orange; BPCX1—blue; IFL—violet.

Liver tissues were collected on the 29th day. As the result of proteomic analysis of 62 extracts, 4617 protein groups were identified (FDR 1%) (Table S5). Evaluation of the proteomic composition in different groups was performed via hierarchical clustering based on the average levels of label-free quantification (LFQ) intensities. ANOVA label-free proteomic analysis revealed 222 proteins (FDR < 0.01) that were significantly changed with respect to the negative control (Table S6). The T-SNE dimensionality reduction method shows the distribution of samples into major clusters (negative control, IFL, other groups) (Figure 4B). To assess the changes in the proteome composition between different treatment groups, a Mann–Whitney test was performed. The proteomic composition of individual liver samples of BALB/c mice with subacute liver failure caused by CCl_4_ allowed us to reliably distinguish intact and IFL samples. Heat map analysis shows that negative control and IFL groups could be clearly distinguished by hierarchical clustering as well (Figure 4A).

**Figure 5:**
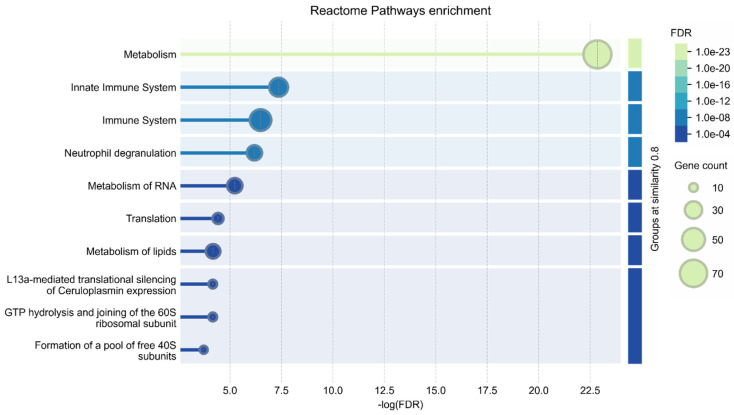
Reactome Pathway GO annotation of 222 significantly changed liver proteins (FDR < 0.01).

Annotation of the 222 significantly changed proteins using the GO database (Figure 5) revealed their participation in xenobiotic metabolic processes (CYP2F2, CYP2A5, AKR1C12, CYP2D22, PON3, VKORC1, CYP2C37, AOX3, CYP2D9, CYP2A4, ABCB11, FMO5), xenobiotic stimulus (21 proteins), and responses to chemical stimuli (50 proteins) associated with response to CCL_4_.

Several proteins were also participants in innate immune system pathways (35 proteins), the adaptive immune system (17 proteins), and responses to oxidative stress (14 proteins); these changes could be associated with SALF progression, while proteins participating in drug distribution, metabolism, and excretion (ADME) (PON1, CYP2D22, NAT1, HPRT, PON3, UGT1A2, SLCO1B2, ITPA) as well as aromatic compound catabolic processes (PON1, CYP2F2, ENDOG, TYMP, HPRT, UROD, CDA, PON3, DCPS, KMO, DERA, ITPA) might be associated with polyphenol intake.

Pairwise comparison revealed 101 proteins that were significantly (*p*-value < 0.5, abs (Cohen’s d-size) < 0.05) changed between the CCL_4_ and negative control groups (Table S7). Most of the proteins participated in the regulation of cell death (21 proteins), responses to chemical stimuli (27 proteins), metabolic processes (71 proteins), and biosynthetic processes (30 proteins).

### 2.5. Results of Molecular Fingerprinting by FT-ICR MS

Fourier transform ion cyclotron resonance mass spectrometry (FT-ICR MS) is well suited for metabolomics research and can provide valuable insights into the molecular fingerprinting of complex biological systems due to its unique capabilities, such as its exceptionally high resolution (>10^6^) and sensitivity, with a mass accuracy of <ppm. In this study, FT-ICR MS was applied to examine if changes in molecular profiles can be associated with the experimental group. Unlike conventional LC–MS/MS metabolomic analysis, such an approach prevents metabolite annotation, but fingerprinting is capable of catching ephemeral changes in molecular composition. For each group, the conservative number of determined molecular formulae (MF) varied from 252 for the ISF group control to 362 MF for the negative control in the acidic fraction. In the fraction isolated at a neutral pH, 176 to 352 MFs were determined for the negative control and HA group, respectively (Table S8).

**Figure 6:**
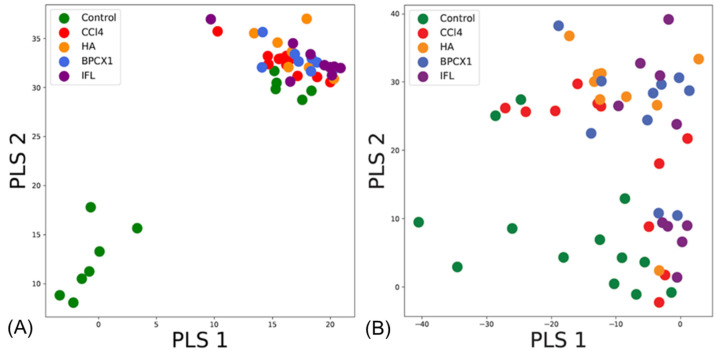
Results of PLS-DA analysis based on FT-ICR MS data obtained for of liver tissues fractions: (**A**) acidic fraction, (**B**) neutral fraction.

Statistical analysis of the two fractions separately provided insights into the similarity of the experimental groups. Application of PLS-DA to the acidic fraction showed a clear distinction of the negative control from the other groups for liver tissue fractions (Figure 6A). At the same time, the neutral fraction (Figure 6B) samples from the negative control and ISF groups clustered together, while other samples did not form any distinct clusters. This was supportive of the proteomics analysis and the highest observed hepatoprotective activity of ISF.

The issues with direct infusion FT-ICR MS analysis include loss of material during the solid-phase extraction and the low reproducibility of relative intensities. This limits the applicability of FT-ICR MS for comparisons of samples. To overcome this limitation, we applied a formulae-difference algorithm that has been proven to be more tolerant to experimental discrepancies even in interlaboratory comparisons [26]. Table S9 contains data on the total number of unique formulae differences in neutral and acidic fractions. Despite the relative content remaining the same in all experimental groups, the absolute number of formulae differences was significantly lower in the CCl_4_ group. The extremely high number of formulae differences in the case of the HA group may indicate better tissue penetration, which corroborates well with the previously reported in vivo hepatoprotective activity of humic substances [27].

Comparisons of groups based on the distribution of formulae differences (Figure S3) revealed the high similarity of the negative control to the therapeutic groups compared to the CCl_4_ group. Although formulae differences lack direct connections to biochemical pathways, they do indicate that the administration of hepatoprotective agent supports the occurrence of metabolic processes, which were suppressed after the addition of CCl_4_.

## 3. Discussion

Proteomic analysis revealed new insights into the molecular mechanisms of the hepatoprotective activity of multicomponent mixtures of natural origin. A significant difference was observed in the proteome profiles of the urine and liver of BALB/c mice with SALF between the negative control, CCl_4_ control, and samples receiving potential hepatoprotectors of natural origin (BP-Cx-1, Ha, IFL). Molecular fingerprinting of metabolites with FT-ICR MS (Table S10) corroborated the proteome results. PCA analysis demonstrated a clear cluster of control samples, while samples from other groups showed a mixed picture (Figure S3). Nevertheless, the ISF group demonstrated higher similarity to the control group, with a cosine similarity of 0.75, which also supports the proteomic results.

The proteins that were significantly changed in urine during CCL_4_ administration are involved in extracellular matrix organization (Comp, Ctsb, Vcam1, Spp1, Ceacam1, Ctsl), as well as in abnormal wound healing (Scpep1, Comp, Tgfbr1, Spp1, Ceacam1). A significant decrease in the level of Osteopontin (SPP1) was observed in the CCl_4_ group. SSP1 is an extracellular matrix protein involved in immune system processes—in particular, type-1 immune responses—through the enhancement of the production of interferon-gamma, interleukin-10, and interleukin-12 cytokine expression. [28]. Prominin-1 (PROM1) is another significantly increased protein involved in cell differentiation, proliferation, and apoptosis [29]. In contrast, the level of cartilage oligomeric matrix protein (COMP) was significantly increased. COMP is a suppressor of apoptosis that blocks the activation of caspase-3 and induces the IAP family of survival proteins [30]. Carcinoembryonic antigen-related cell adhesion molecule 1 (CEACAM1) plays the role of a co-inhibitory receptor in the immune response, affects the action of insulin, and also functions as an activator of angiogenesis [31,32,33]. Such changes can be associated with the acute-phase response and the release of proteins from the liver, which act as inflammatory mediators and scavengers during tissue repair. The decreasing of apoptotic and immune proteins in treatment groups can be related to the restoration of the inflamed tissue back to a normal status.

The most pronounced changes were observed in the IFL group—40 significantly changed proteins were measured with respect to the CCl_4_ and negative control groups. Those proteins participate in the regulation of apoptosis (Ctsb, Plg, Cxadr, Ltf, Thbs1), innate immune system processes (Ctsb, Vtn, Hexb, Plau, Ltf, Ceacam1, Ear1, Creg1, Ctsl), as well as the organization of the extracellular matrix (Comp, Ctsb, Plg, Vtn, Vcam1, F11r, Thbs1, Fn1, Ceacam1, Col15a1, Ctsl). Cathepsin B (CTSB) protein levels were increased after CCl_4_ administration and decreased after IFL treatment. CTSB is a thiol protease that is involved in intracellular protein and extracellular matrix degradation [34]. Plasmin (PLG) was another protein that decreased with respect to the CCL_4_ group. PLG acts as a proteolytic enzyme in a variety of processes including tissue remodeling and inflammation—in particular, in neutrophil apoptosis, macrophage reprogramming, and efferocytosis [35].

**Figure 7:**
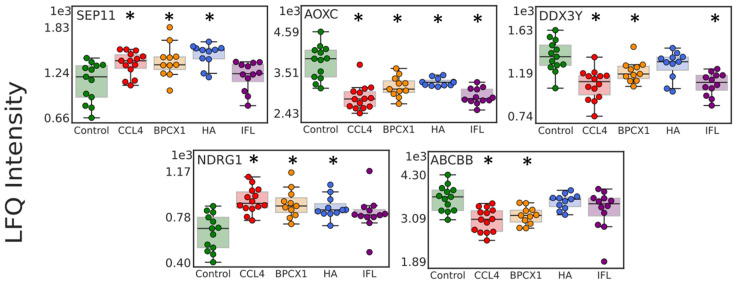
Boxplots for 5 significantly changed proteins (AOXC, NDRG1, ABCBB, DDX3Y, SEP11) suggested as potential targets for drug intervention. * indicates a significant difference at the *p*-value < 0.05 and the absolute value of Cohen’s d effect size > 0.5.

Among the 101 significantly changed in mouse liver proteins, five proteins (AOXC, NDRG1, ABCBB, DDX3Y, SEP11) (Table S7) could be suggested as potential targets for drug intervention, based on their average concentration between the CCL_4_ and control groups (Figure 7). It is worth noting that NDRG1 is a stress-responsive protein involved in hormone responses, cell growth, and differentiation, and acts as a tumor suppressor in many cell types. NDRG1 also participates in p53/TP53-mediated caspase activation and apoptosis [36]. Another important protein target is septin 11 (SEP11), which is involved in the regulation of reactive oxygen species homeostasis and mediates the 27-hydroxycholesterol activation of endothelial inflammation via estrogen receptor alpha [37].

According to the results of a proteomic analysis, the greatest hepatoprotective effect is exerted by the use of IFL, which correlates well with the relative effectiveness of the drugs recorded in in vitro studies.

Unlike IFL, BPCx-1 and HA are extremely complex mixtures. This complexity prevents the accurate determination of their active components. Still, in our previous study, we demonstrated [20] that isolation of the least-polar fraction from BP-Cx-1 increases its hepatoprotective activity. It was also reported that isolation of the least-polar fraction of humic acids results in a higher non-specific affinity to the proteins [38]. Likely, antioxidation properties explain the hepatoprotective activity of all agents under study. Therefore, fractionation of HA and BP-Cx-1 and increasing the contribution of polyphenols would increase their efficiency

These results correlate with the relative effectiveness of the drugs recorded in previous in vitro and in vivo studies and may be due to the lower molecular weight of the active isoflavonoids compared to the components of the polyphenolic drug BP-Cx-1 [39,40].

## 4. Materials and Methods

### 4.1. Chemicals

1. Fraction of humic acids (HA) from low-mineralized silt sulfide mud (peloids) of lake Molochka, Sergievsky district, Samara region, Russia (MRC “Sergievskie Mineral Waters” FMBA of Russia).
2. Fulvic acid (FA) of low-mineralized silt sulfide mud (peloids) of lake Molochka, Sergievsky district, Samara region, Russia (MRC “Sergievskie Mineral Waters” FMBA of Russia).
3. Castoreum extract (CE). Light orange powder [41]; 100 g of raw material contains 46.6 g of beaver musk extract (FSUE NPC “Pharmzashchita” FMBA of Russia). For in vitro study, powder of beaver musk substance was diluted in distilled water until a homogeneous suspension was formed. Sample weight 0.499 g. An alcohol extract was prepared by adding ethyl alcohol in a weight ratio of 1:2; the solution was treated with ultrasound and centrifuged at 13,000 rpm to remove the sediment. The solution was evaporated and re-dissolved in a nutrient medium.
4. Isoflavones from kudzu Pueraria lobata roots (IFL)—kudzu root extract (Shaanxi Sheng, Xi’an, China) [42]. Sample weight 0.82 g. An alcohol extract was prepared by adding ethyl alcohol in a weight ratio of 1:2; the solution was treated with ultrasound and centrifuged at 13,000 rpm to remove the sediment. The solution was evaporated and redissolved.
5. Water-soluble lignin derivative BP-Cx-1 [3] (Nobel Ltd., Saint Petersburg, Russia)—a sterile 0.42% ammonia solution (batch X0621D33). In the in vitro studies, BP-Cx-1 was tested at a concentration of 0.0042% (V/V), and for the in vivo studies, at a concentration of 0.42% (V/V).

### 4.2. Animal Studies

All animal studies were carried out at the center for preclinical research of the Federal State Institution “National Medical Research Center of Oncology named after N.N. Petrov” of the Ministry of Health of the Russian Federation in accordance with the Study Protocol and standard operating procedures.

The study cohort comprised 62 female BALB/C mice obtained from the Stolbovaya branch of the FSBSI “Scientific Center for Biomedical Technologies of the Federal Medical and Biological Agency” of Russia. Before the start of the experiment, animals underwent quarantine and adaptation for 17 days. Mice were housed at a room temperature of 20–23 °C, a relative air humidity of 54–58%, and an air exchange rate of 8 volumes per hour. All animals received standard complete briquetted chow (Laboratorkorm, Moscow, Russia) and filtered water ad libitum. Mice were examined daily by the veterinarian.

### 4.3. Experimental Design

Mice were randomized by weight and the following groups of animals were formed:

1. Control—negative control—intraperitoneal injection of sunflower oil (0.2 mL/mouse) (*n* = 13).
2. Control CCl_4_—positive control—intraperitoneal injection of CCl_4_ (1 mL/kg) diluted ten times in sunflower oil 2 times per week (6 injections in total) (*n* = 14).
3. CCl_4_ + Fraction of humic acids (HA)—intraperitoneal injection of CCl_4_ (1 mL/kg) diluted ten times in sunflower oil 2 times per week (6 injections in total) + intragastric administration of HA (60 mg/kg) 3 times a week, for 4 weeks (*n* = 12).
4. CCl_4_ + BP-Cx-1—intraperitoneal injection of CCl_4_ (1 mL/kg) diluted ten times in sunflower oil 2 times per week (6 injections in total) + intragastric administration of BP-Cx-1 (60 mg/kg) 3 times a week, for 4 weeks (*n* = 11).
5. CCl_4_ + isoflavones isolated from the root of Pueralia lobata (IFL)—intraperitoneal injection of CCl_4_ (1 mL/kg) diluted ten times in sunflower oil 2 times per week (6 injections in total) + intragastric administration of IFL (60 mg/kg) 3 times a week, for 4 weeks (*n* = 12).

The CCl_4_ intake concertation were pre-established at a 1 mL/kg concentration during the pilot study in accordance with Scholten et al. [24]. The CCl_4_ concentration (1 mL/kg) was well tolerated and induces hepatotoxicity in in the BALB/C mice model 28 day after the first administration.

Body weighing was carried out twice a week during experiments and prior to euthanasia.

Urine samples were collected using metabolic cages (Tecniplast, Buguggiate, Italy) for 18 h a day before euthanasia. To obtain the analyzable volume, urine samples pulled from two mice were transferred to Eppendorf tubes and kept frozen at −80 °C.

Euthanasia by CO_2_ was carried out on the 29th day after the first administration of CCL_4_. All animals were autopsied. The liver was excised and weighted, and the mass index (percentage ratio of liver mass to body weight) was calculated. Liver samples were transferred to Eppendorf tubes, immediately frozen in liquid nitrogen, and kept frozen at −80 °C for further analysis. The rest were histologically examined after routine histological preparation (dehydration, impregnation with paraffin, cutting into sections, staining with hematoxylin and eosin). Evaluation of liver steatosis was carried out microscopically in 10 fields (×200) per slide by a semi-quantitative method (in points): 0—no changes, 1—up to 30% of hepatocytes contain lipid vacuoles in the cytoplasm, 2—30–60% of cells are changed, 3—more than 60% of cells are changed. The mean steatosis index was calculated per animal and onward per group.

No deterioration in the condition of the nature-derived compound-treated animals was detected, which may indicate the absence of a pronounced toxic effect of the compositions in the current model.

### 4.4. MTT Colorimetric Assay

The MTT colorimetric assay was used to determine the cell cytotoxicity and hepatoprotective activity of multicomponent mixtures of polyphenolic compounds on HepG2 hepatocyte cell cultures. The HepG2 was obtained from the ATCC cell bank (HB-8065). Cells were cultured in DMEM (PanEco company, Moscow, Russia) supplemented with 10% fetal bovine serum (Thermo Fisher Scientific, Waltham, MA, USA), 2 mM L-glutamine (Thermo Fisher Scientific, USA), 100 U/mL penicillin (Sigma, Berlin, Germany), and 100 μg/mL streptomycin (Sigma, Germany). Incubation was carried out at 37 °C in 5% CO_2_. In order to determine the hepatoprotective activity of the multicomponent mixtures, HepG2 cells were incubated (5 × 10^3^ cells/well; 96-well plates, for 24 h) with 1 mmol CCl_4_ (LenReactiv, Saint Petersburg, Russia) in 0.15% DMSO for inducing apoptosis [43]. After 24 h incubation, cell cultures were washed twice with a PBS buffer. Next, potential hepatoprotectors were added at an initial concentration of each drug per well of 1 mg/mL, with a dilution step of 1/2, and incubated for 24 h. Cell cultures were washed in a PBS buffer and 20 μL of MTT (Sigma, Germany) was added to each well and incubated for another 2 h. After incubation, the medium was removed from the plates and 100 μL of DMSO was added to each well to dissolve the formed formazan crystals. Cell viability was identified by measuring the optical density with an ELISA reader (Tecan Infinite M200 PRO, Mannedorf, Switzerland) at 630 nm.

### 4.5. Proteomic Analysis

#### 4.5.1. Urine Sample Preparation

Urine samples were denatured and reduced by incubation with 8 M urea, 0.1 M dithiothreitol, and 100 mM Tris × HCl (pH 8.0, +37 °C, 30 min). Next, the proteins were alkylated by a 30 min incubation in the dark with 55 mM iodoacetamide. Protein concentration was measured using the Bradford method. For trypsinolysis, the samples were diluted with 100 mM Tris × HCL (pH 8.0) until <1 M urea; L-(tosylamido-2-phenyl) ethyl chloromethyl ketone (TPCK)-treated trypsin (Worthington) was added at a 25:1 (protein:enzyme, *w*/*w*) ratio; and the samples were incubated for 16 h at 37 °C. The reaction was quenched by acidifying the samples with formic acid (FA) to a final concentration of 1.0% (pH ≤ 2). The resulting peptides were purified by solid-phase extraction on C18 cartridges (Oasis, Waters, San Antonio, TX, USA), then lyophilized and dissolved for analysis in 0.1% formic acid.

#### 4.5.2. Liver Sample Preparation

Liver tissue (10–20 mg) was ground into a fine powder in liquid nitrogen. Cell lysis was conducted by adding 50 mM Tris-HCl (pH 8.0), 150 mM NaCl, 0.1% SDS, 0.5% Na deoxycholate, and 1% NP-40 lysis buffer containing a cocktail of protease inhibitors (Roche, Basel, Switzerland). Tissues were incubated for 30 min and sonicated in an ultrasonic bath twice for 3 min. Samples were centrifuged at 40 °C at 10,000× *g* for 10 min, the supernatant was collected, and the sediment was re-extracted with denaturing buffer (8 M urea, 2 M thiourea, 50 mM Tris-HCl pH 8.0, 0.5% NP-40). The supernatant was collected and pooled after the centrifugation at 40 °C at 10,000× *g* for 10 min. Protein concentrations were measured by the Bradford method; 100 μg of protein was taken and precipitated with ice-cold acetone. Samples were denatured and reduced by incubation with 8 M urea, 0.1 M dithiothreitol, and 100 mM Tris × HCl (pH 8.0, +37 °C, 30 min). Next, the proteins were alkylated by a 30 min incubation in the dark with 20 mM iodoacetamide. For trypsinolysis, the samples were diluted with 100 mM Tris × HCl (pH 8.0) until <1 M urea; L-(tosylamido-2-phenyl) ethyl chloromethyl ketone (TPCK)-treated trypsin (Worthington) was added at a 25:1 (protein:enzyme, *w*/*w*) ratio; and the samples were incubated for 16 h at 37 °C. The reaction was quenched by acidifying the samples with formic acid (FA) to a final concentration of 1.0% (pH ≤ 2). The resulting peptides were purified by solid-phase extraction on C18 cartridges (Oasis, Waters, USA), then lyophilized and dissolved for analysis in 0.1% formic acid.

#### 4.5.3. LC–MS/MS Analysis

Urine tryptic peptide fractions were analyzed on a nano-HPLC (high-performance liquid chromatography) Dionex Ultimate 3000 system (Thermo Fisher Scientific, USA) coupled to a timsTOF Pro (Bruker Daltonics, Billerica, MA, USA) mass spectrometer in three technical replicates. HPLC separation was carried out using a packed emitter column (C18, 25 cm × 75 μm 1.6 μm) (Ion Optics, Parkville, Australia), with gradient elution. Mobile phase A was 0.1% formic acid in water; mobile phase B was 0.1% formic acid in acetonitrile. LC separations were performed at a flow of 400 nL/min, using a 40 min linear gradient from 2% to 37% solvent B, followed by an LC column wash step (12 min isocratic with 90% solvent B) and equilibration (15 min, isocratic, with 2% solvent B). The MS data were acquired using the ddaPASEF method. Electrospray source (ESI) settings were as follows: capillary voltage 1500 V, dry gas flow—3.0 L/min at a temperature of 180 °C. The MS and MS/MS spectra were acquired from 100 to 1700 m, an ion mobility range of 0.6–1.6 1/K0 (V×s/cm^2^). The ion mobility was scanned from 0.6 to 1.6 V×s/cm^2^. The ramp time was set to 100 ms. The collision energy was ramped linearly as a function of the mobility from 59 eV at 1/K0 = 1.6 V×s/cm^2^ to 20 eV at 1/K0 = 0.6 V×s/cm^2^.

Tissue tryptic peptide LC separations were performed at a flow of 400 nL/min using a 90 min linear gradient from 2% to 37% solvent B, followed by an LC column wash step (10 min isocratic with 90% solvent B) and equilibration (15 min, isocratic, with 2% solvent B) in three technical replicates. The MS data were acquired using the diaPASEF method. Electrospray source (ESI) settings were as follows: capillary voltage 1400 V, dry gas flow—3.0 L/min at a temperature of 180 °C. The MS and MS/MS spectra were acquired from 100 to 1700 m, an ion mobility range of 0.6–1.6 1/K0 (V×s/cm^2^). The ion mobility was scanned from 0.6 to 1.6 V×s/cm^2^. The ramp time was set to 100 ms. The collision energy was ramped linearly as a function of the mobility from 59 eV at 1/K0 = 1.6 V×s/cm^2^ to 20 eV at 1/K0 = 0.6 V×s/cm^2^.

#### 4.5.4. Data Analysis

The obtained LC–MS/MS data were analyzed using PEAKS Studio v. 11 software (BSI, North Waterloo, ON, Canada), using the following parameters: parent ion mass measurement error—20 ppm; fragment mass error—0.05 Da. An unspecific restriction search for peptides 6–45 amino acids long was conducted across the UniprotKB Mus Musculus database with carbamidomethylation (C) and oxidation (M) as possible modifications. FDR thresholds were set to 0.1% at the PSM level and 1% at the protein group level, with the requirement of at least one unique and one significant peptide identification. A Peak library (.tsv) for the diaPASEF date search was generated by ddaPASEF acquisition of the mouse liver pooled samples.

The statistical analysis and data visualization were performed by Python (3.7.3) with the following packages: SciPy [44], Seaborn [45], Matplotlib [46], and Pandas [47]. Significant differences in protein concentrations in the mouse groups were estimated using the Mann–Whitney U-test. We considered concentration values to be statistically significant if the *p*-value was less than 0.05 and the absolute value of Cohen’s d effect size was greater than 0.5. Heat-map hierarchical clustering and principal component analysis (PCA) with t-distributed stochastic neighbor embedding (t-SNE) were used for preliminary estimation of the differences between the studied groups and particular samples. For visualizing the clustering of mice in a single group, we used the UMAP dimensionality reduction algorithm [48]. For Lasso regression, the Scikit-Learn [49] package was used, and all data were preprocessed with z-scoring. The default value was chosen for parameter C, and all possible pairwise comparisons were conducted between all groups. Training was performed on the entire dataset.

### 4.6. Molecular Fingerprinting by FT-ICR MS

#### 4.6.1. Extraction of Metabolites

For metabolites analysis, a chloroform–methanol extraction method was used [50]. Samples were mixed with a chloroform–methanol (1:2) mixture and incubated for 30 min with constant stirring; after this, samples were sonicated twice in an ultrasonic bath. Next, water and chloroform were added up to ratio of 1/2/0.8 and centrifuged at 3000 rpm. The upper water–methanol phase was evaporated via a centrifugal vacuum evaporator and purified using PPL cartridges. Metabolites were eluted sequentially with 0.5 mL of MeOH (Neutral fraction) and 0.5 mL of MEOH/0.5% FA (Acidic fraction). Neutral and acidic fractions were dried in a centrifugal vacuum evaporator and reconstituted in 50% MeOH-H_2_O before analysis.

#### 4.6.2. FT-ICR MS Analysis

Mass spectrometric analysis of metabolites was carried out using a FT-ICR MS Apex Ultra device (Bruker Daltonics), with a harmonized cell equipped with a 7 T superconducting magnet and electrospray (ESI) ionization source. All mass spectra were acquired in negative ionization mode by direct infusion at a flow rate 90 mL/h. The electrospray source (ESI) settings were as follows: capillary voltage—3500 V, dry gas flow—3 (N2) l/min at a temperature of 200 °C. Measurements were carried out in the mass/charge (*m*/*z*) range of 200 to 1000. Spectra were acquired at 400,000 resolutions (at *m*/*z* 400), with 250 scans accumulated for each spectrum.

#### 4.6.3. Data Analysis

Raw FT-ICR MS data were treated using open-source software and lab-written Python scripts. Visualization of data has been performed with Python library Matplotlib [46]. Statistical analysis has been performed with the Python libraries NumPy, Pandas, and Seaborn. All *.raw files were converted to *.mzML format using msconvert with a continuous wavelet transform algorithm to extract peaks with an S/N > 3 [51]. Peak lists were extracted using a Python script based on the pyteomics library [52]. Formulae assignment for all samples was conducted with a Python script based on the open-source NOMspectra Python library [53], with a denoising step adapted from Zielinski et al. [54], using the following constraints: O/C ratio ≤ 1, 0.3 < H/C ratio < 2.5; element counts [1 < C ≤ 60, 2 < H ≤ 100, 0 < O ≤ 60, N ≤ 3, S ≤ 1]; z = −1 and mass accuracy window < 1 ppm after build-in internal calibration based on the construction of the probability density of assignments [55]. High-resolution mass spectrometry data classification based on the differences in the number of elements between each pair of molecular formulae within the formulae list was conducted using open source software Nommass https://nommass.com (accessed on 10 March 2023) [26].

In the next step, molecular assignments that presented in at least three samples of each group were concatenated into a new group-representative table, giving aggregated data. This step aimed to decrease the uncertainty of the qualitative comparison raised from the unknown mass balance. Further statistical analysis and evaluations were conducted similarly to the proteomic data.

## 5. Conclusions

According to the results of a previous study, BP-Cx-1 and IFL show a pronounced hepatoprotective effect. In this study, we applied proteomic and metabolomics research to examine how the addition of BP-Cx-1 and HA affects metabolic pathways in mice with steatosis. Both complex mixtures showed moderate in vitro and weak in vivo activity, but their impact on the composition of the proteome and the biochemical reactions in mice was noticeable. The isolation of more active fractions can be a next step.

## Data Availability

Data are contained within the Supplementary Materials.

## Supplementary Materials

The supporting information can be downloaded at: https://www.mdpi.com/article/10.3390/ijms26041604/s1.
