# Supplementary material for: Hepatoprotective Activity of Nature-Derived Polyphenols Studied by Mass Spectrometry Based Multi-OMICS Approach
Source: Int J Mol Sci. 2025 Feb 13;26(4):1604. doi: 10.3390/ijms26041604 (PMC11855638; doi:10.3390/ijms26041604)
Supplement: Supplementary file 1 [file ijms-26-01604-s001.zip › Supplementary Figures_Academic Editor Notes_Feb02_AGB.pdf]

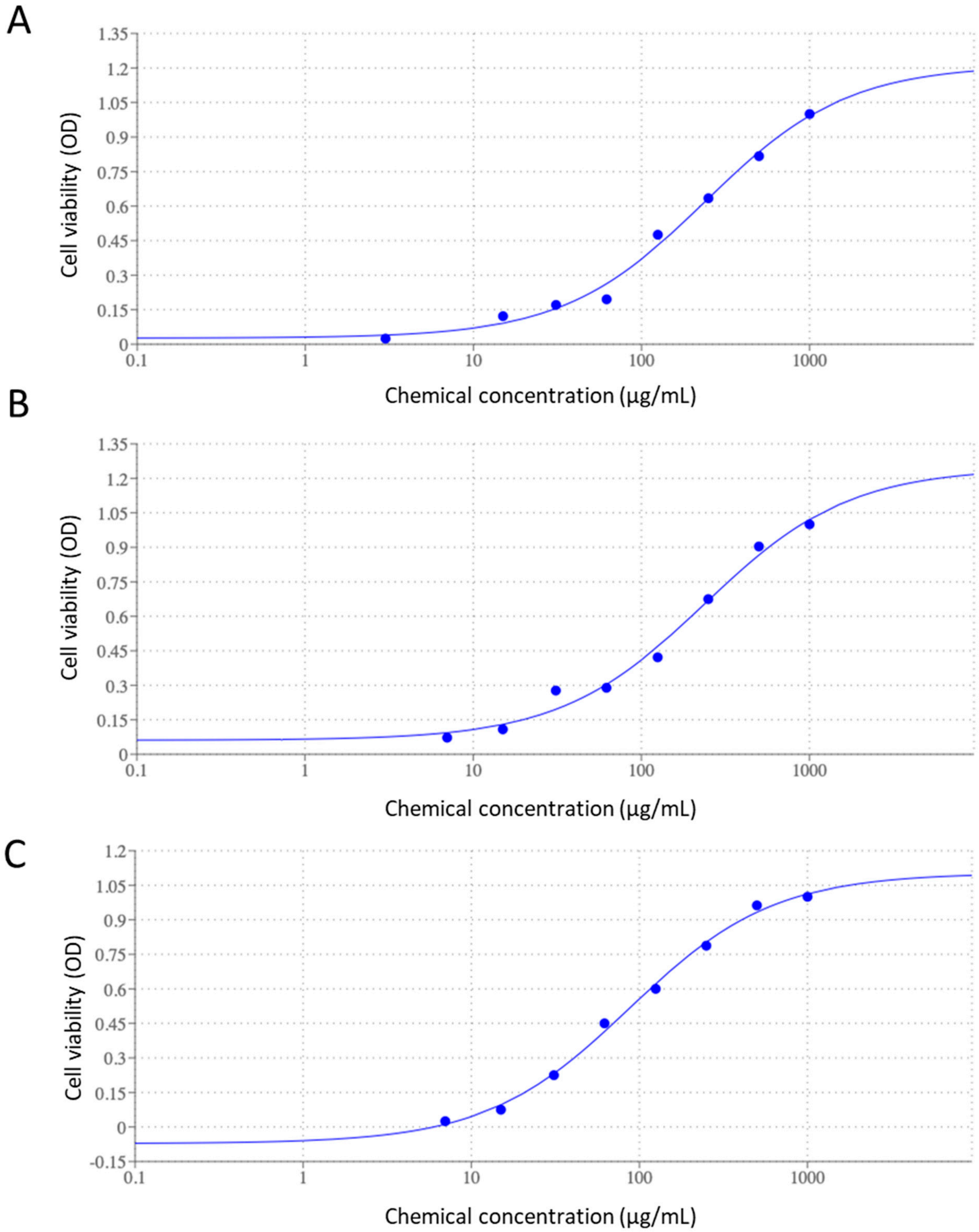

Supplementary Figure S1. Calculation of the effective dose 50 (ED50) using linear-logarithmic interpolation via AAT Bioquest online calculator: (A) ED50 GK = 238.9997 µg/ml; (B) ED50 BP-Cx- 1 = 236.9988 µg/ml; (C) ED50 isoPL = 87.1657 µg/ml. Optical density (OD) is plotted along y – axis and chemicals concentrations (µg/mL) along x – axis.

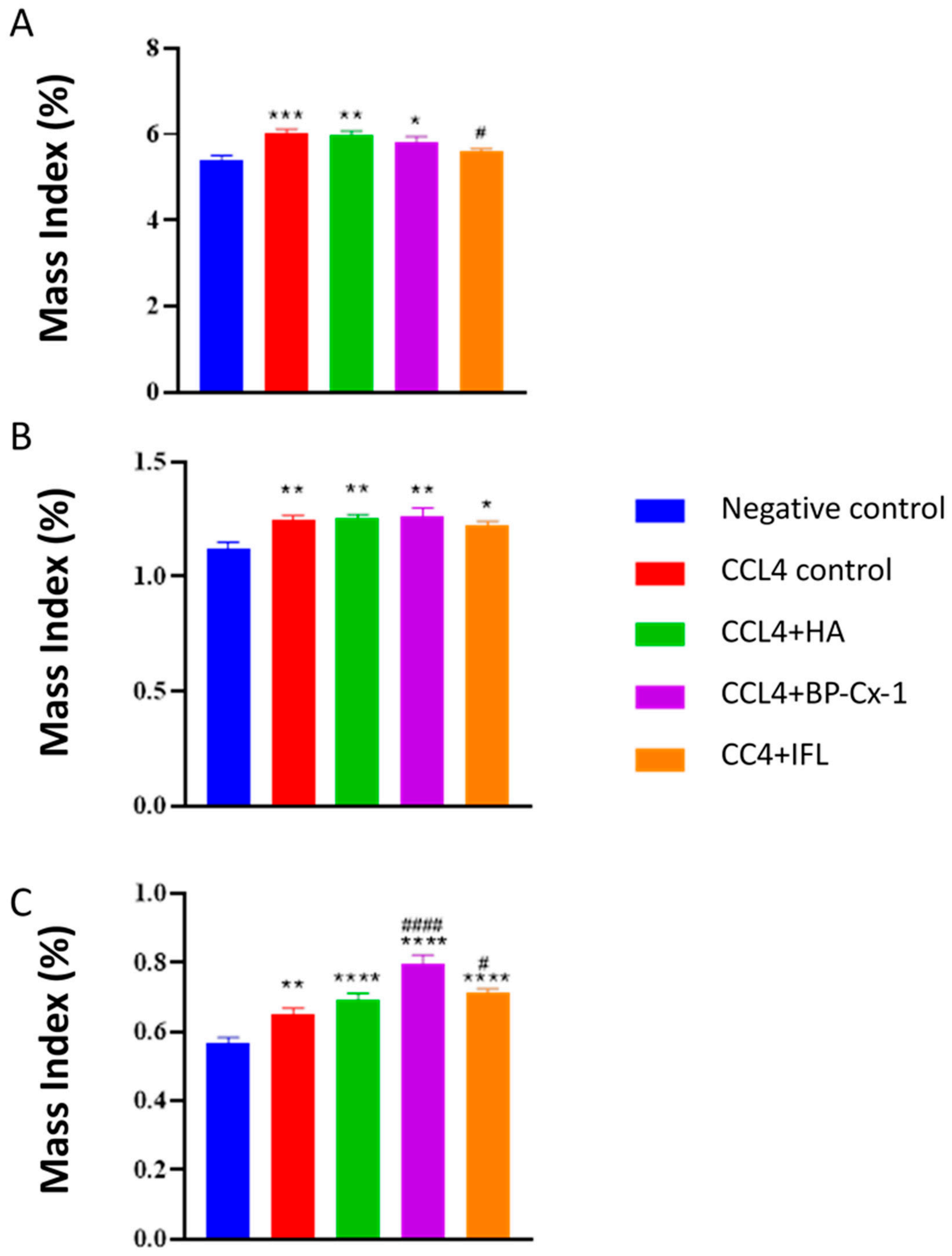

Supplementary Figure S2. Comparison of internal organs mass indexes (percentage ratio of organ mass to body weight) in the BALB/C mice model with SALF induced by the toxin carbon tetrachloride (CCL4): (A) liver, (B) kidneys, (C) spleen. * marker indicates a statistical significant difference (**** - p-value <0.0001, *** - p-value <0.001, ** - p-value <0.01, * - p-value <0.05) with respect to the Control group. # marker indicates a statistical significant difference (#### - p-value <0.0001, # - p-value <0.05) with respect to the CCL4 group.

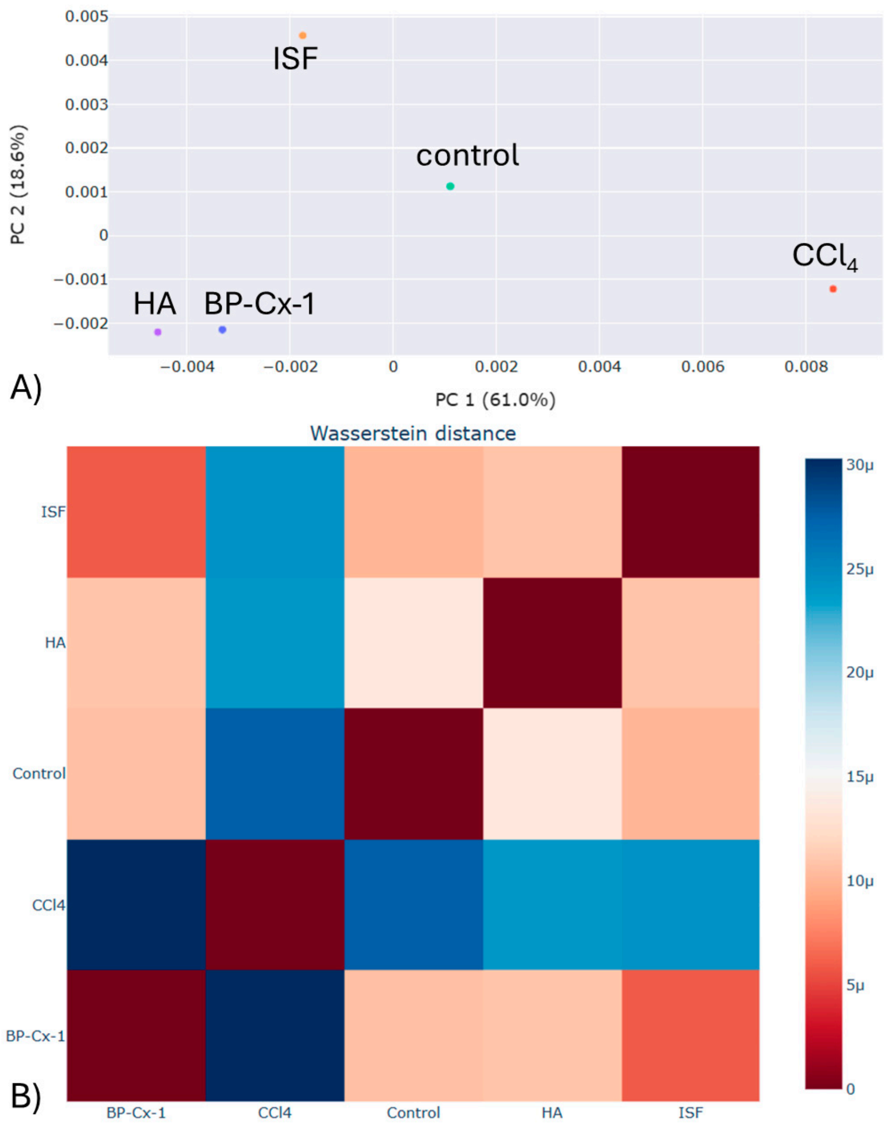

Supplementary Figure S3. A) PCA biplot, B) Wasserstein distance based on formulae-difference statistics for experimental groups
